# Supplementary material for: Upscaling of integrated photoelectrochemical water-splitting devices to large areas
Source: Nat Commun. 2016 Sep 7;7:12681. doi: 10.1038/ncomms12681 (PMC5023961; doi:10.1038/ncomms12681)
Supplement: Supplementary Information — Supplementary Figures 1-15 and Supplementary Methods. [file ncomms12681-s1.pdf]

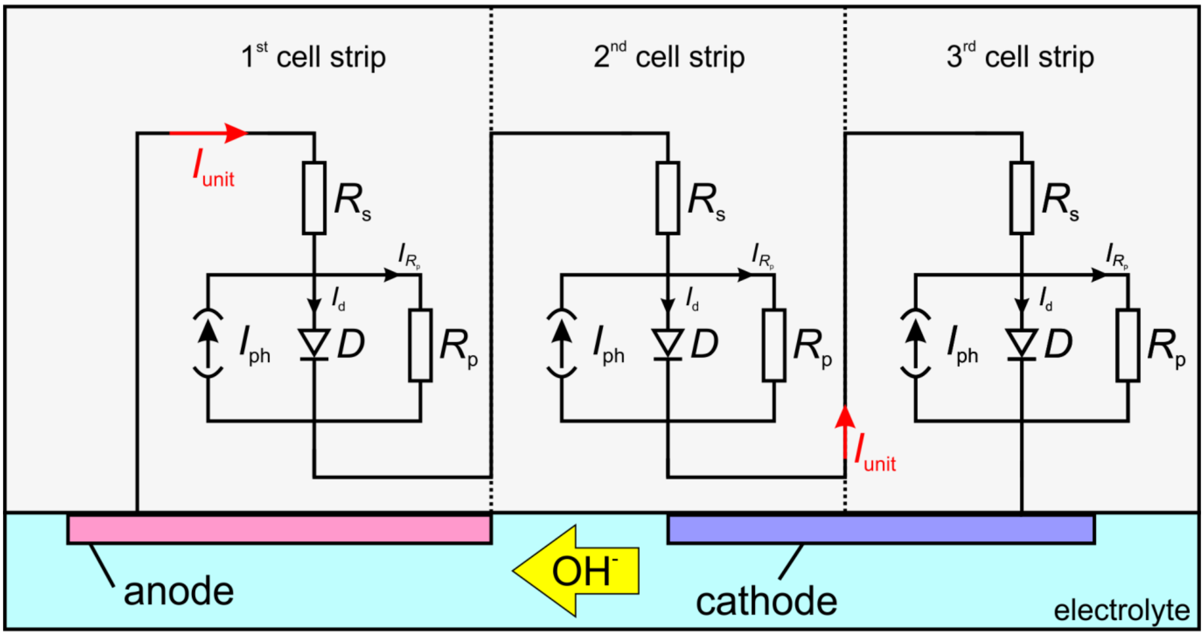

Supplementary Figure 1 | Electrical circuit sketch. Equivalent circuit diagram of the singular base unit device #2 consisting of three single junction thin-film silicon a-Si:H solar cells connected in series. The current through the unit is depicted by I_{unit}. The current loop is closed by the ion flow through the electrolyte.

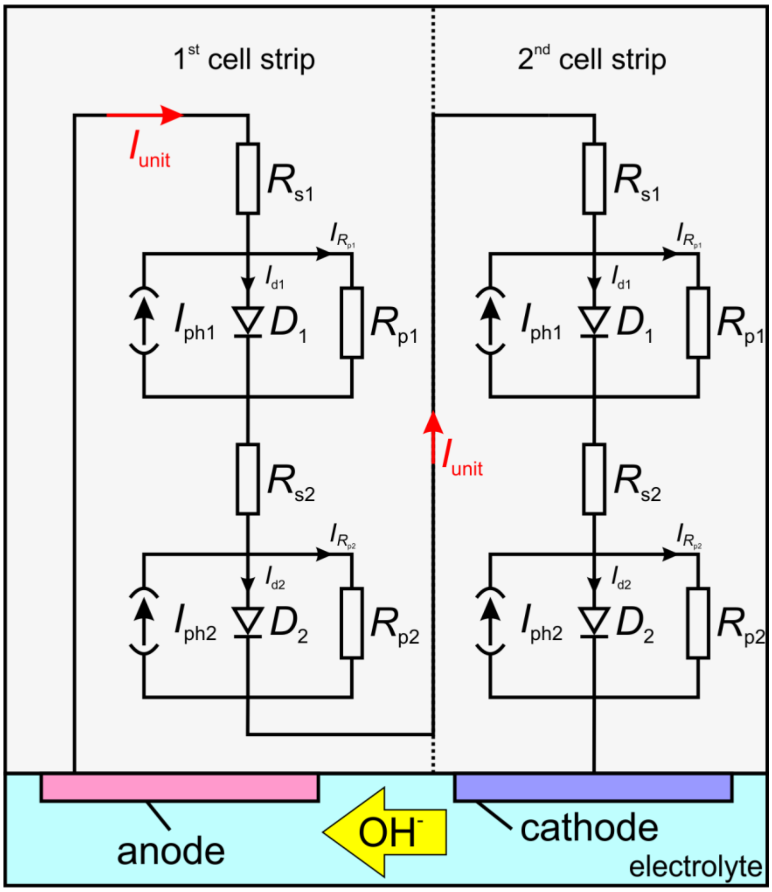

Supplementary Figure 2 | Electrical circuit sketch. Equivalent circuit diagram of a singular base unit as in device #3 consisting of two multi-junction thin-film silicon a-Si:H/µc-Si:H solar cells connected in series. The individual sub cells are depicted by the indices 1 and 2 for the top- and bottom-cell, respectively. The current through the unit is depicted by I_{unit}. The current loop is closed by the ion flow through the electrolyte.

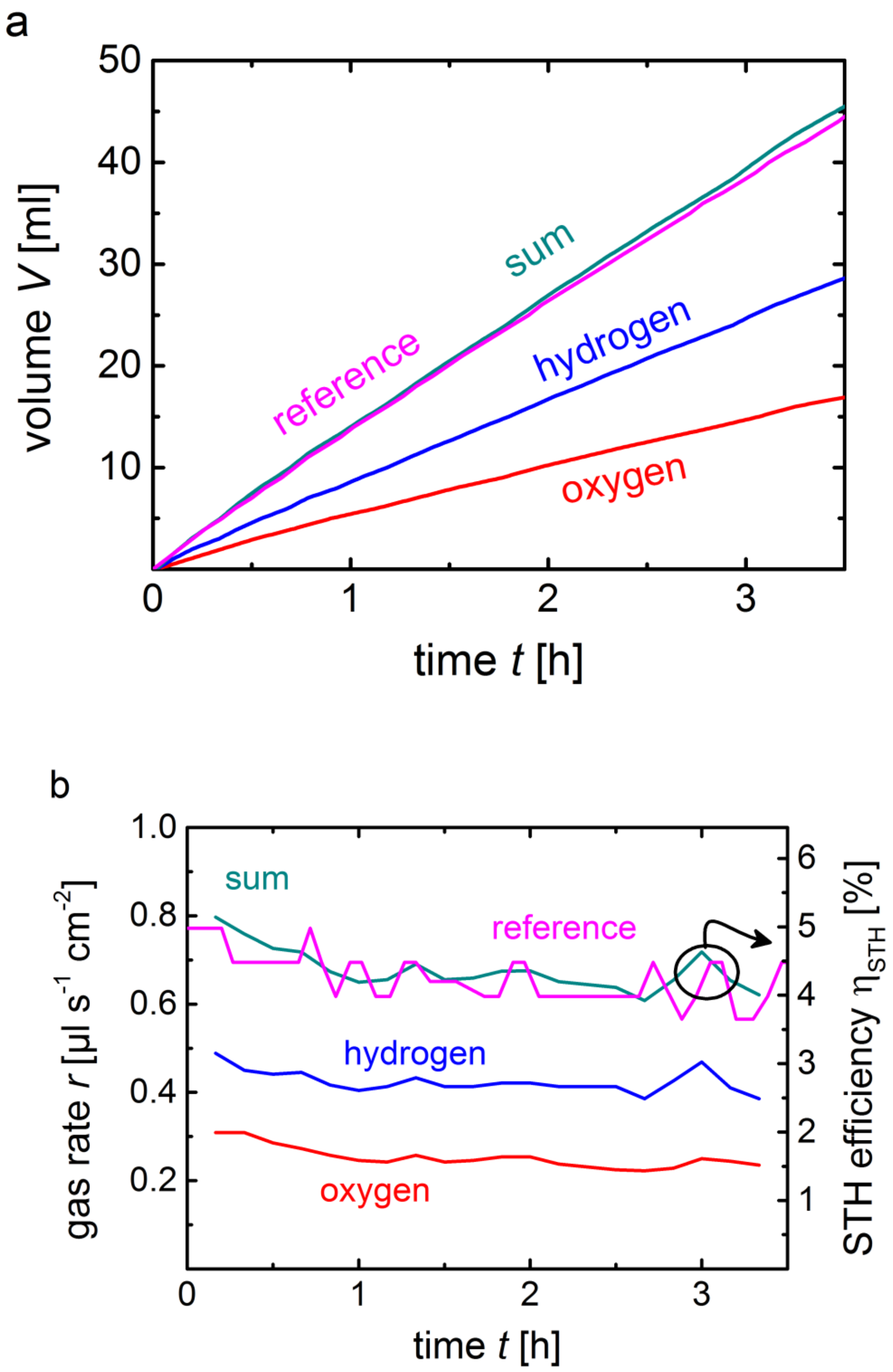

Supplementary Figure 3 | Membrane tests. Collected gas volume (**a**) and gas evolution rate (**b**) of two single base unit devices with anion exchange membrane between cathode and anode. A second reference device is shown with the same geometry and comparable *I-V* data but without a membrane where both gases were co-collected. The right hand axis in (**b**) depicts the STH efficiency for the sum of both gases and the reference, respectively.

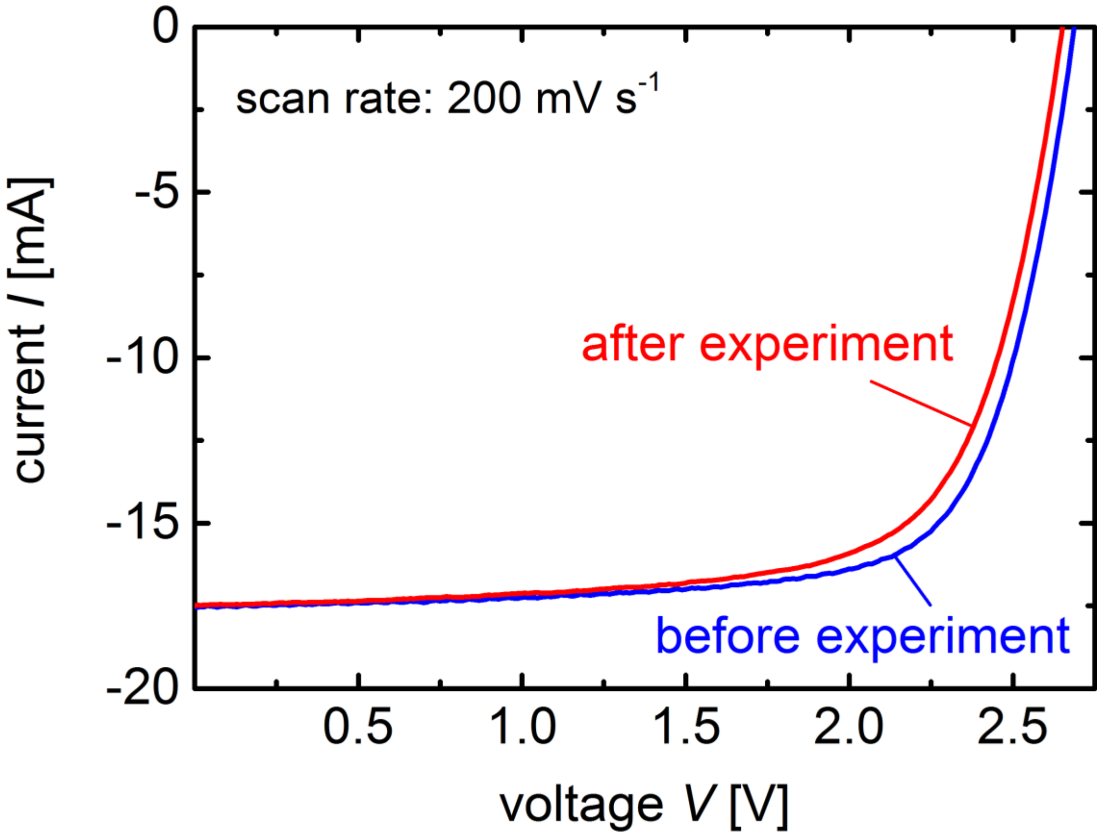

Supplementary Figure 4 | PV element degradation. Plot of the PV element´s *I*-*V* characteristics of device #1 before and after 3 hours of operation under illumination. A reduction of the fill-factor and a lowered open-circuit voltage can be seen from the curve after operation. Device degradation as well as increased temperatures could be responsible for such behavior since there was no control over the device temperature. Both measurements start from $V = 0$ V and a scan rate of 200 mV s^{-1} was employed.

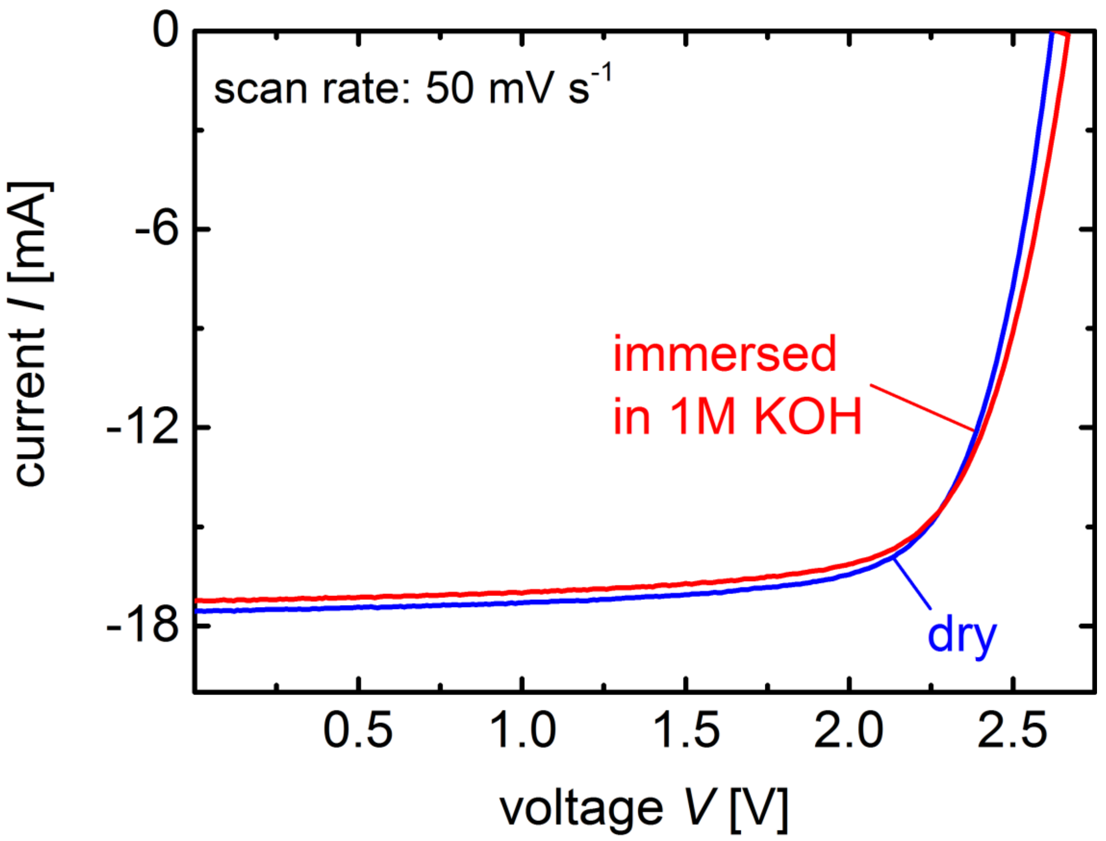

Supplementary Figure 5 | Effect of electrolyte immersion. Plot of the PV element's *I*-*V* characteristics of device# 1 dry and immersed in 1M KOH before operation. Both curves have differences in short-circuit current as well as in the open-circuit voltage. One explanation for these differences can be identified by a change of the device temperature by cooling due to the electrolyte which acts as a heat sink to a certain degree. Both measurements start from $V = 0$ V and a scan rate of 50 mV s^{-1} was employed.

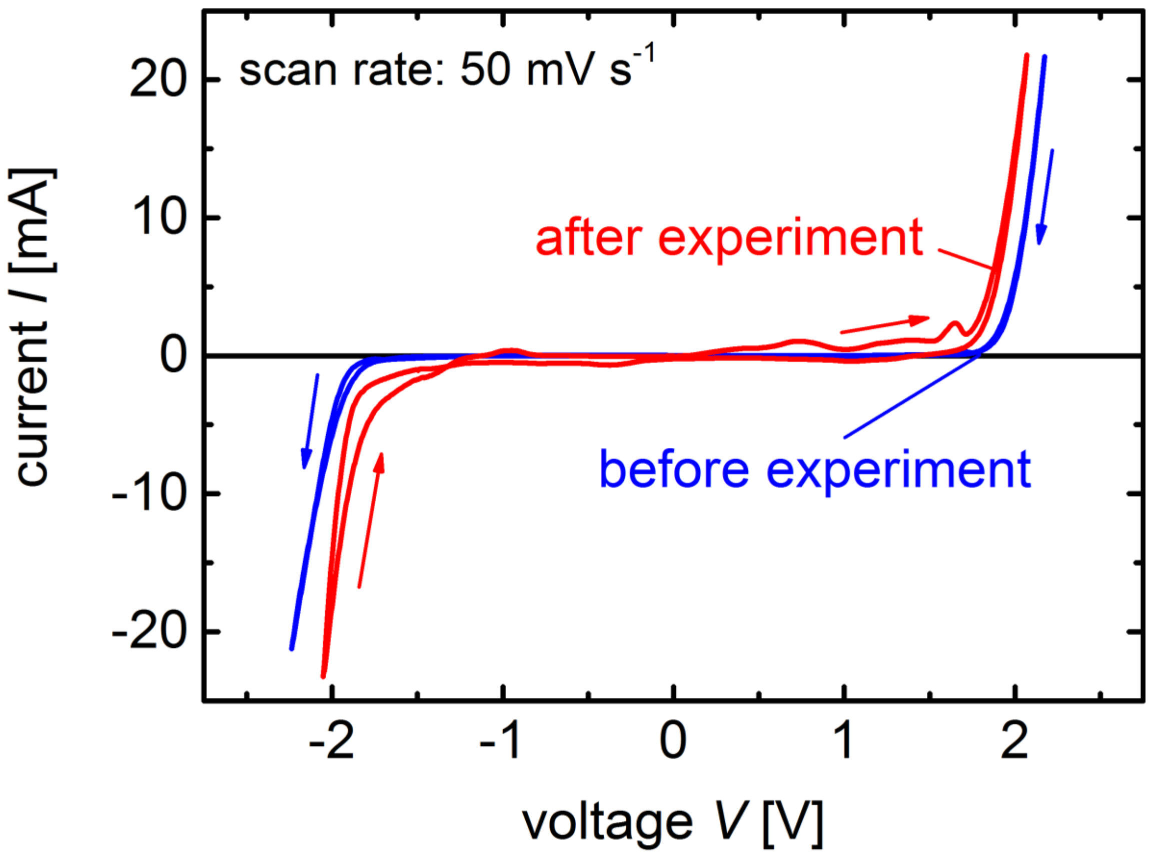

Supplementary Figure 6 | Change of EC element properties. Comparison of the EC element´s *I-V* properties from device #1 before and after 3 hours of operation under illumination in 1M KOH. After operation a lower overpotential is observed which can be explained be chemical changes of the nickel-foam surface. Oxidation and reduction potentials can be identified that were initially not apparent. A scan rate of 50 mV s^{-1} was employed. Please note that no preconditioning of the nickel-foam was done prior to the experiment.

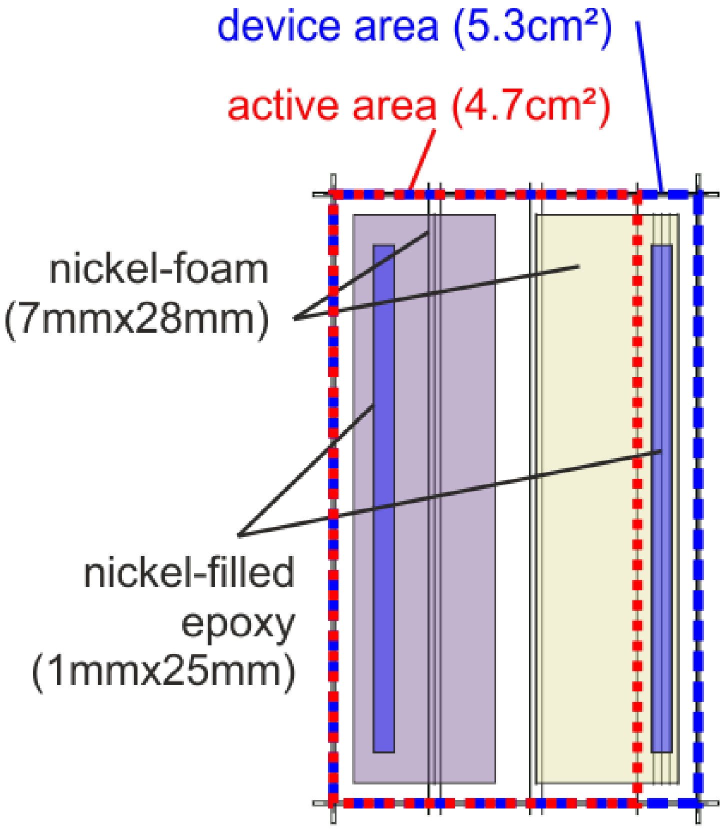

Supplementary Figure 7 | Device geometry. Schematic sketch of the laser design file for the patterning process of device #2. A transparent overlay indicates the position and size of the EC element´s anode and cathode on the back side. The areas depicted by the red and blue dashed rectangles define the active and device area, respectively. This distinction is important since the difference between both areas, due to the 2 mm wide fillet to the front contact, was required for practical reasons during device manufacture. The active area losses due to the interconnection region were neglected.

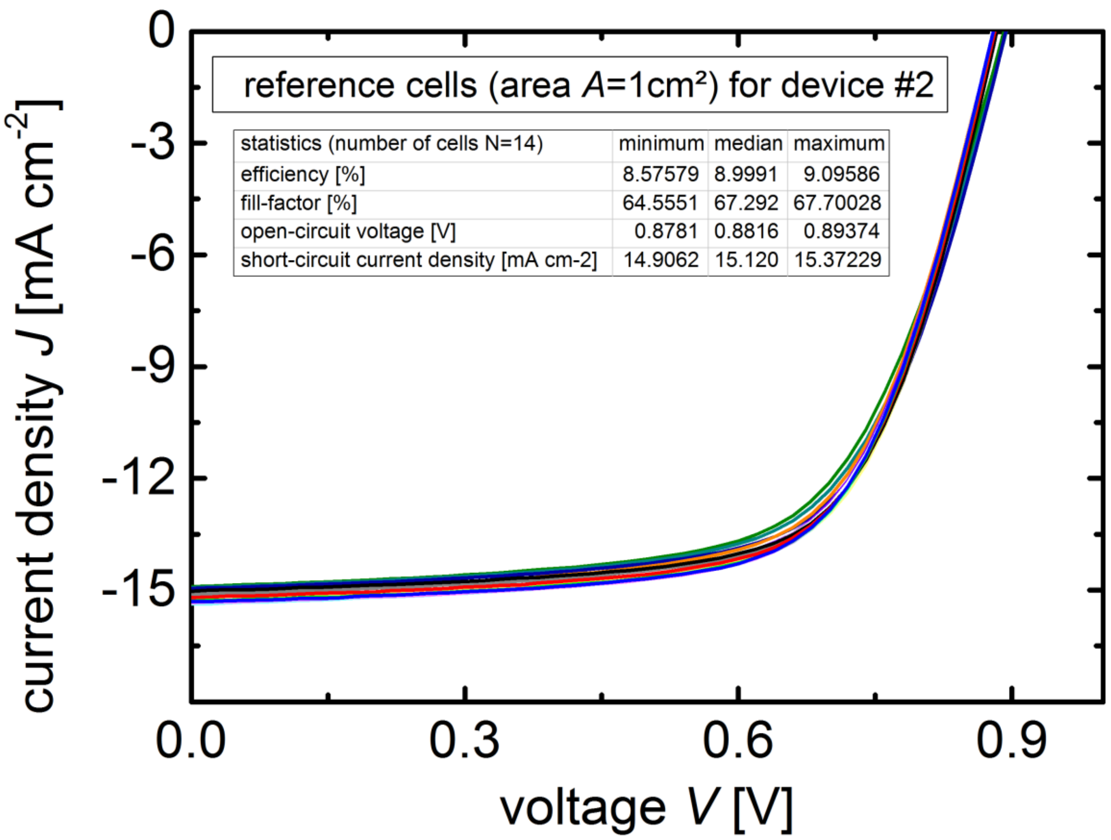

statistics (number of cells N=14)	minimum	median	maximum
efficiency [%]	8.57579	8.9991	9.09586
fill-factor [%]	64.5551	67.292	67.70028
open-circuit voltage [V]	0.8781	0.8816	0.89374
short-circuit current density [mA cm-2]	14.9062	15.120	15.37229

Supplementary Figure 8 | Reference solar cells. Solar cell *J*-*V* properties of reference a-Si:H solar cells (area *A* = 1 cm²) that were deposited in the same deposition sequence as the stability test base unit named device #2. A total of 14 solar cells were measured on a $\mathbf{10\ cm} \times \mathbf{10\ cm}$ substrate. The statistics in the inset table show only minor variations of the solar cell properties.

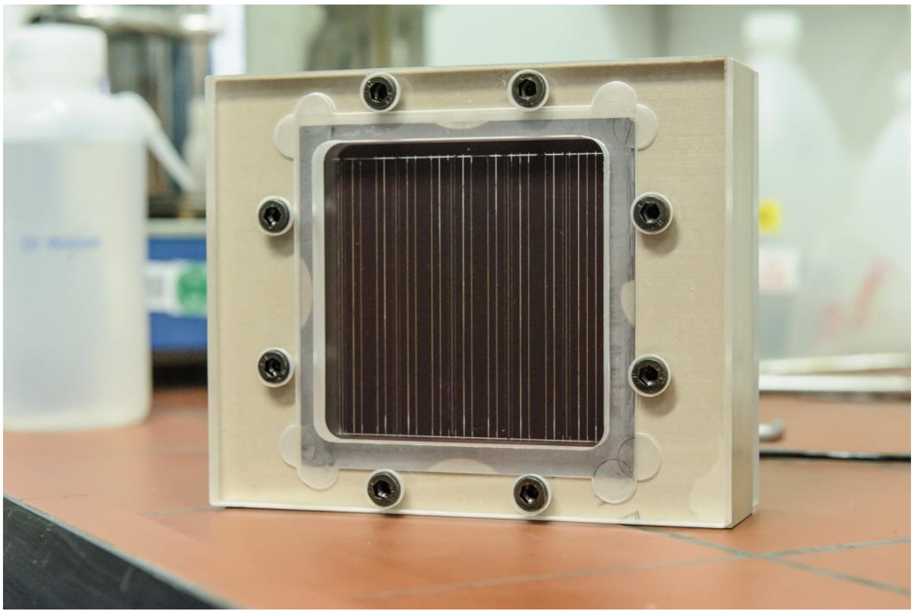

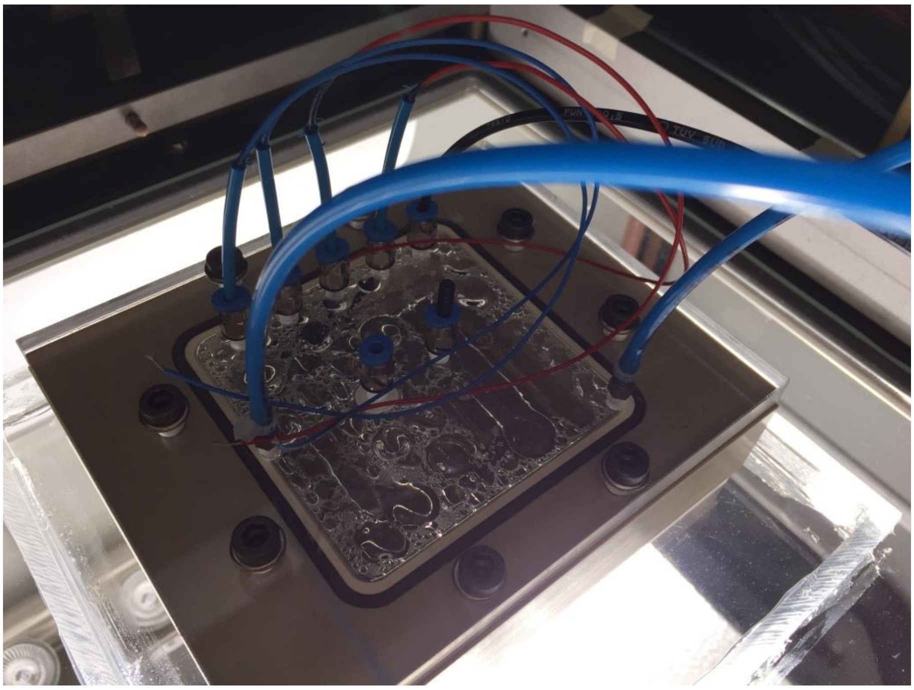

Supplementary Figure 9 | Large scale devices. (Top) Photograph of the sample holder shown from the front side assembled with the upscaled $\mathbf{10\ cm} \times \mathbf{10\ cm}$ module ($\mathbf{8\ cm} \times \mathbf{8\ cm}$ aperture area) similar to device #3. (Bottom) Photograph of the measurement setup for the upscaled water splitting devices with an in-house built large area sun simulator. The device was illuminated from the bottom. Voltage monitoring was not applicable for this device.

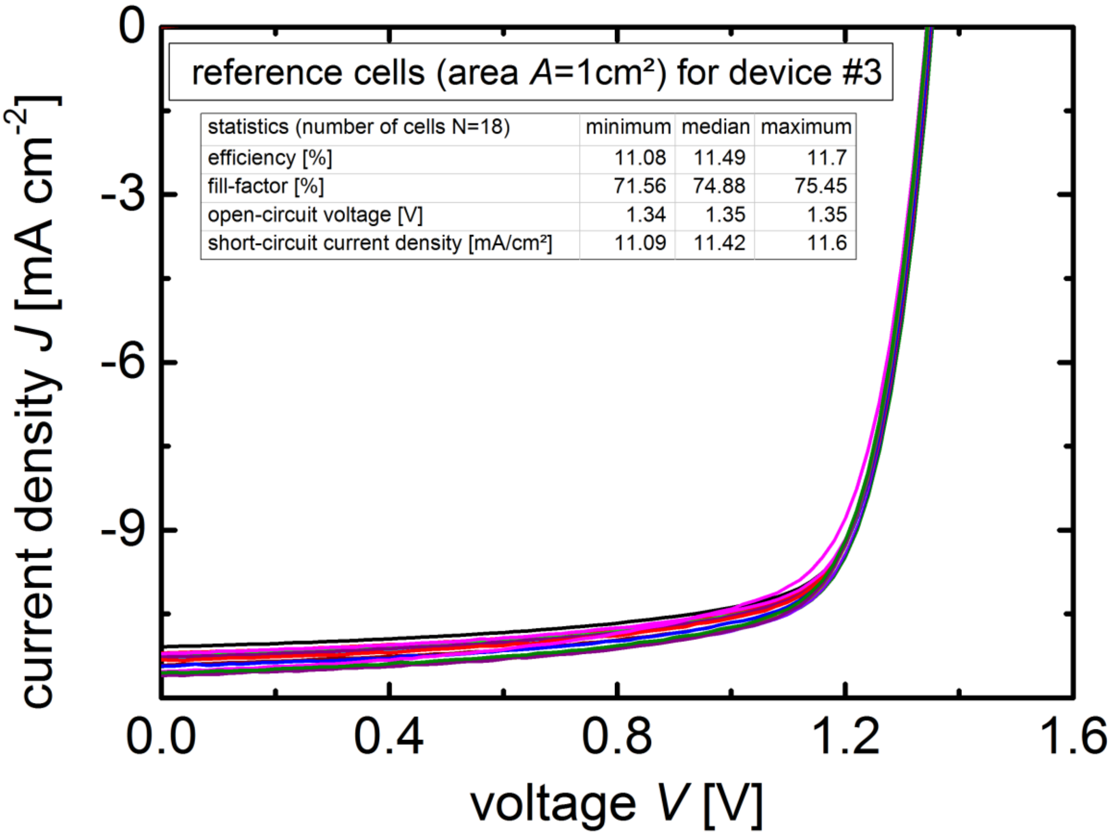

Supplementary Figure 10 | Reference solar cells. Solar cell *J*-*V* properties of single A = 1 cm² reference cells that were deposited in the same run as the upscaled water splitting device #3. A total of 18 solar cells were measured on a $\mathbf{10\ cm} \times \mathbf{10\ cm}$ substrate. The statistics in the inset table show only minor variations of the solar cell properties.

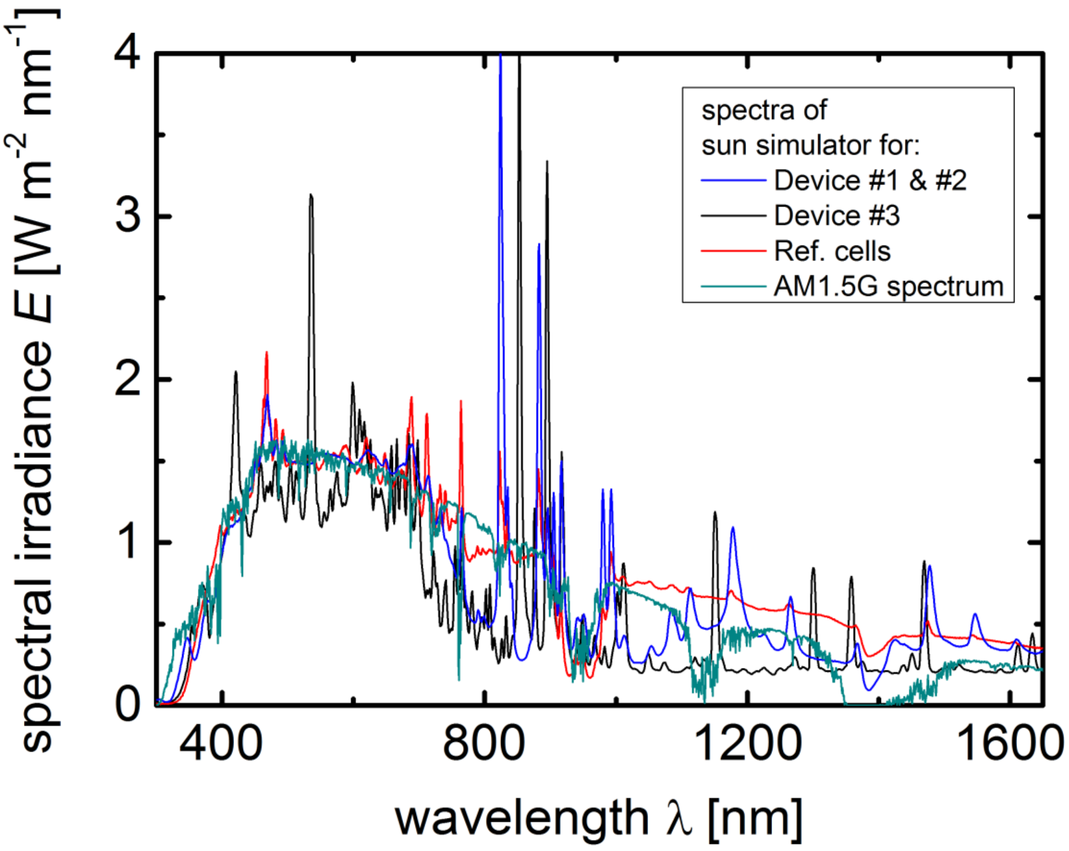

Supplementary Figure 11 | Sun simulator spectra. Plot of spectral power density vs. wavelength of different sun simulators that were used for water splitting experiments and reference solar cell measurements. All illumination sources were steady state lamps. Shown in turquoise is the AM1.5G reference spectrum. There is a good agreement observed between the reference and the sun simulators for the relevant wavelength region of thin-film silicon (400 nm to 1100 nm).

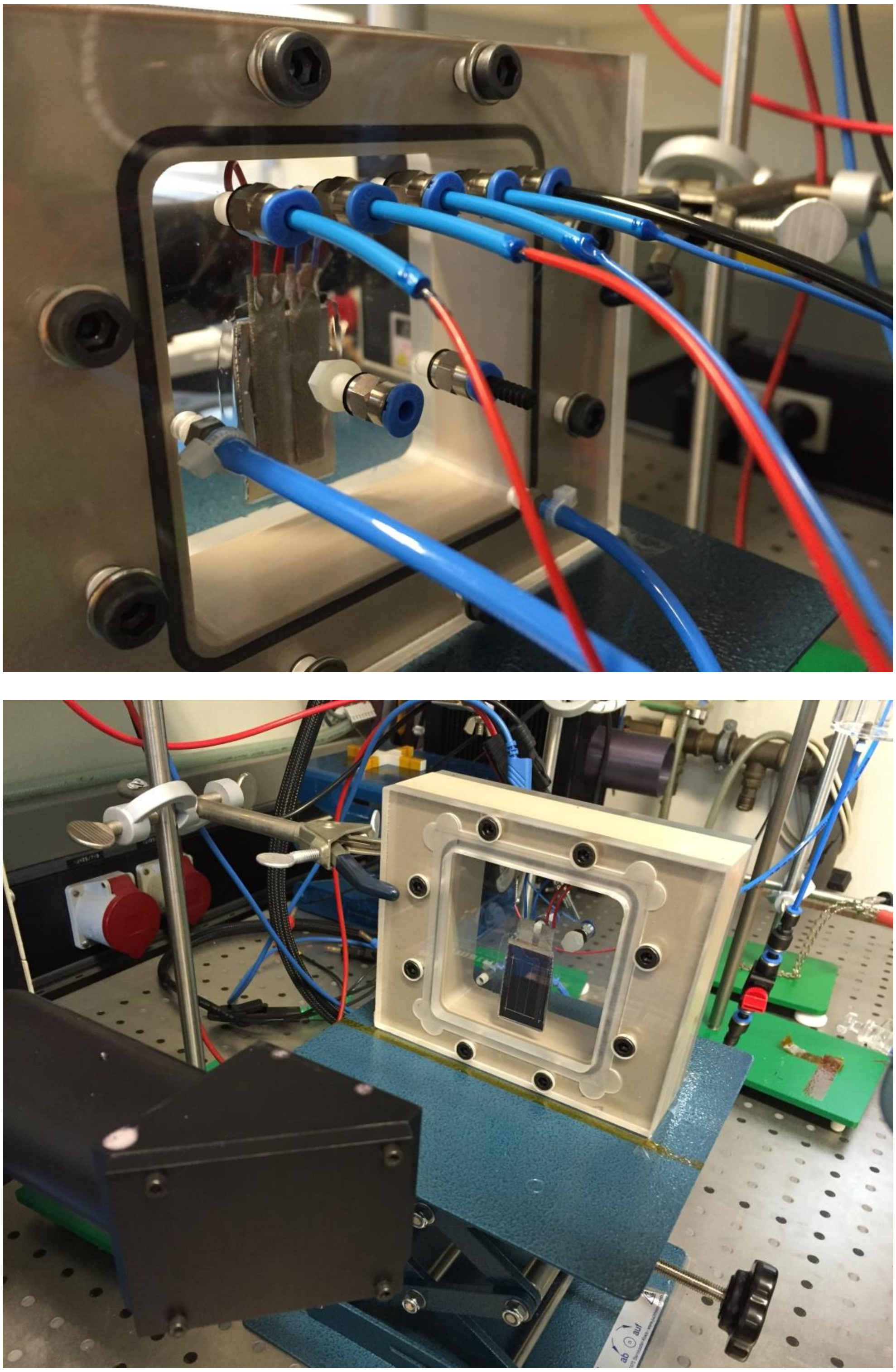

Supplementary Figure 12 | Measurement setup. (Top) Photograph of the measurement setup with the holder made from polyether ether ketone (PEEK) and the single base unit called device #2. Wires were soldered to the anode and cathode for four-wire sensing and fed-through with a gas-tight sealing (blue connectors). Co-evolved reaction products were extracted by the tube in black. The two thicker tubes in blue were used for filling and draining of the electrolyte. (Bottom) Photograph of the setup from the front side with the sun simulator (cf. blue curve in Supplementary Figure 11). The device under test was glued with a transparent epoxy onto a glass substrate for an easy, gas-tight fixture onto the holder.

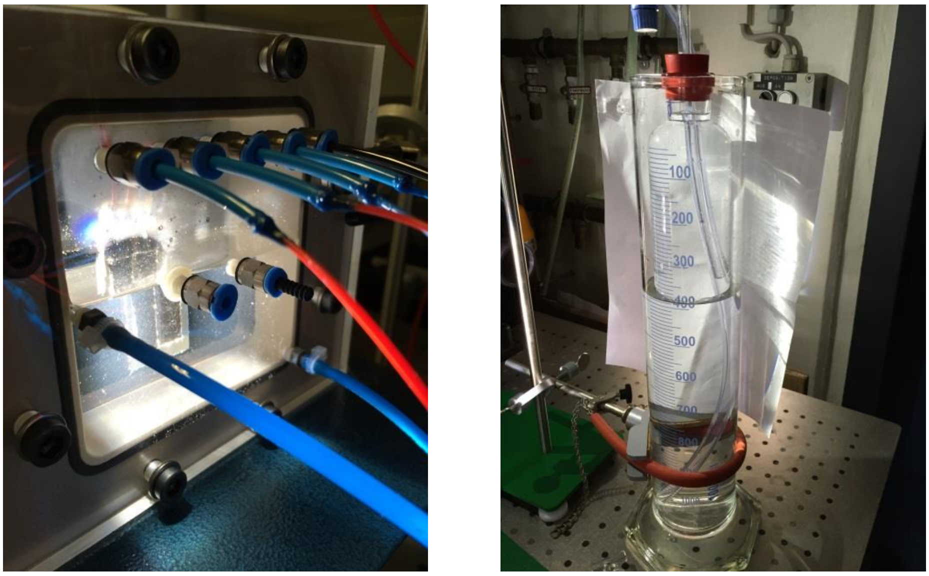

Supplementary Figure 13 | Holder and Bell jar. (Left) Measurement setup with the single base unit device #2 fixed into the sample holder under illumination from the front side. The fixture was filled with 1M KOH as electrolyte. (Right) Photograph of a 1000 ml bell jar (or inverted burette) for collection of the co-evolved gas products (see black tube in the left picture). The gas rate was evaluated by time-lapse photography of the fluid level change over time.

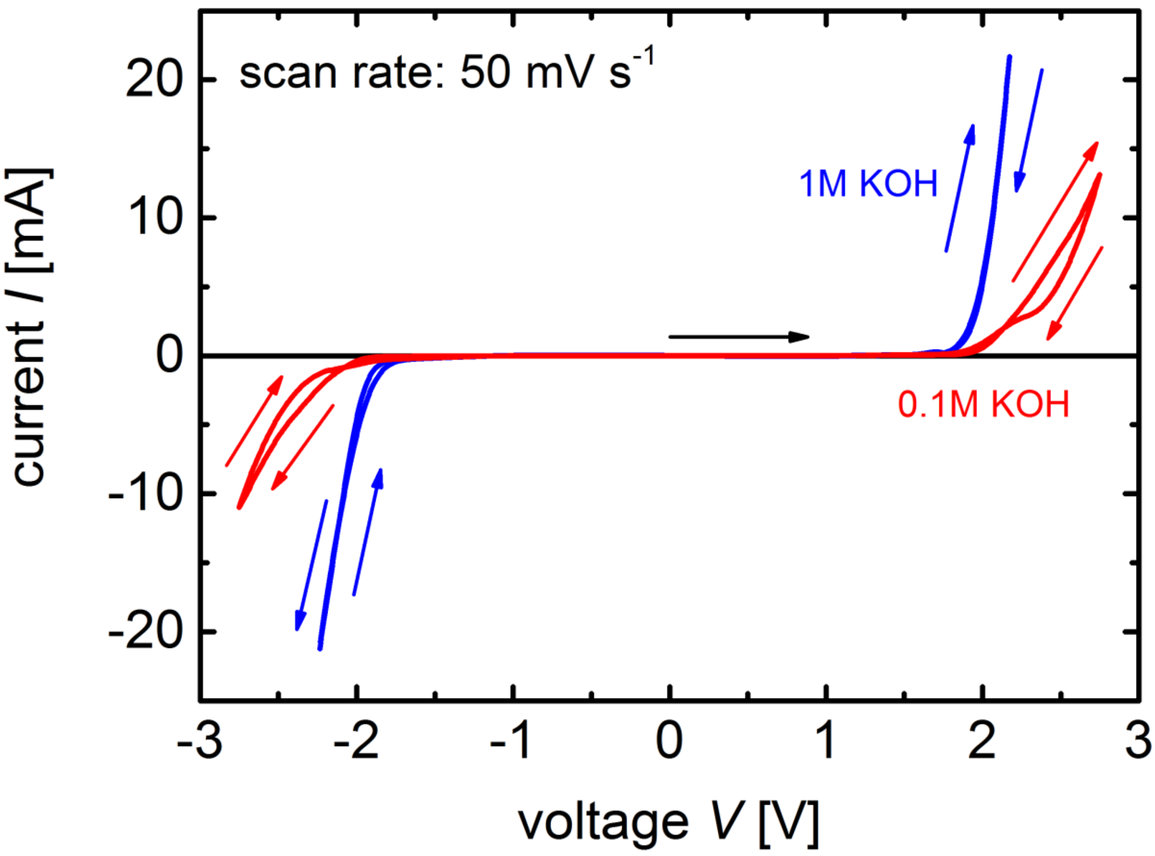

Supplementary Figure 14 | Influence of the KOH concentration. The graph shows the EC element´s *I*-*V* characteristics of device #1. The arrows indicate the scan direction. Both measurements start with V = 0 V into positive voltages (see black arrow). The effect of the higher ionic conductivity of the measurement in 1M KOH solution is clearly visible from the much steeper slope above 1.8 V. A scan rate of 50 mV s^{-1} was employed.

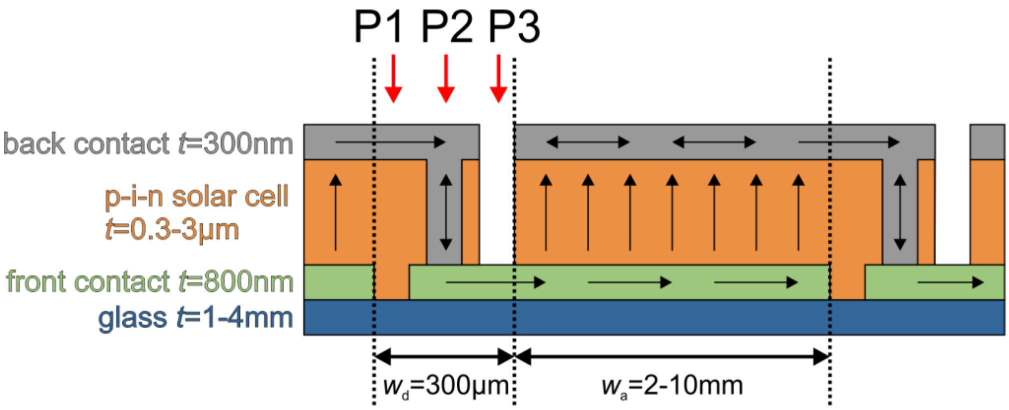

Supplementary Figure 15 | Monolithic series connection. Cross section illustration of a series connected thin-film solar cell in superstrate configuration. The arrows indicate the current flow direction through the solar module. The spatial width required for interconnection structure is defined by w_d while the active solar cell stripe is specified by the width w_a.

Supplementary Methods

Laser processing

Almost every thin-film photovoltaic technology makes use of laser processing for the series connection of solar cells. The series connection is required to lower the ohmic losses in the contacts. We often refer to it as the so called integrated series connection because the required laser processing steps are integrated in between the solar cell layer deposition steps. A first laser process is required after the front contact deposition for cell stripe definition (called P1). After the silicon deposition a second laser processing step is required to selectively remove the absorber, exposing the underlying front contact (P2). Finally, in a last process step the back contact is locally removed by the laser for the final definition of the cell stripes (P3). Supplementary Figure 155 shows an illustration of a single solar module cell stripe with the interconnection structure in cross section.

The individual layers are removed selectively by laser irradiation through the glass substrate leading to an ablation of the material in scribe lines, parallel across the whole substrate. For the P1 process we used a Q-switched DPSS Nd:YVO_4 with a wavelength of 355 nm (third harmonic) and a pulse duration between 7-10 ns. The other two ablation processes P2 and P3 were realized with the use of a similar laser source but with a wavelength of 532 nm (secondary harmonic). This wavelength is favorable since silicon is highly absorbing in this spectral range while the front contact is highly transparent ensuring selective removal without severe damages. The area required for the series connection is no longer active for charge carrier generation and for thin-film silicon typically an area of 3-5% is lost (ratio $f_d = w_d/(w_a + w_d)$). The additional laser processes are required for the fabrication of the presented device concept used similar patterning parameters as the P2 and P3 processes. For practical reasons the fillet to the front contact was 2 mm wide since manual patterning of the insulation epoxy was required (cf. Supplementary Figure 7).
